# The Role of the JAK/STAT Signaling Pathway in the Pathogenesis of Alzheimer’s Disease: New Potential Treatment Target

**DOI:** 10.3390/ijms24010864

**Published:** 2023-01-03

**Authors:** Marta Rusek, Joanna Smith, Kamel El-Khatib, Kennedy Aikins, Stanisław J. Czuczwar, Ryszard Pluta

**Affiliations:** 1 Department of Pathophysiology, Medical University of Lublin, 20-090 Lublin, Poland; 2 Laboratory of Ischemic and Neurodegenerative Brain Research, Mossakowski Medical Research Institute, Polish Academy of Sciences, 02-106 Warsaw, Poland

**Keywords:** Alzheimer’s disease, JAK/STAT signaling pathway, neuroinflammation, neuroprotection, treatment

## Abstract

Alzheimer’s disease is characterized by the accumulation of amyloid plaques and neurofibrillary tangles in the brain. However, emerging evidence suggests that neuroinflammation, mediated notably by activated neuroglial cells, neutrophils, and macrophages, also plays an important role in the pathogenesis of Alzheimer’s disease. Therefore, understanding the interplay between the nervous and immune systems might be the key to the prevention or delay of Alzheimer’s disease progression. One of the most important mechanisms determining gliogenic cell fate is the Janus kinase/signal transducer and activator of transcription (JAK/STAT) signaling pathway that is influenced by the overactivation of microglia and astrocytes. The JAK/STAT signaling pathway is one of the critical factors that promote neuroinflammation in neurodegenerative diseases such as Alzheimer’s disease by initiating innate immunity, orchestrating adaptive immune mechanisms, and finally, constraining neuroinflammatory response. Since a chronic neuroinflammatory environment in the brain is a hallmark of Alzheimer’s disease, understanding the process would allow establishing the underlying role of neuroinflammation, then estimating the prognosis of Alzheimer’s disease development and finding a new potential treatment target. In this review, we highlight the recent advances in the potential role of JAK/STAT signaling in neurological diseases with a focus on discussing future research directions regarding novel therapeutic approaches and predictive biomarkers for Alzheimer’s disease.

## 1. Introduction

Alzheimer’s disease creates a huge burden on the healthcare system and caregivers due to the lack of causal treatment and final etiology. The disease is the main cause of irreversible disability and dementia worldwide. Alzheimer’s disease occurs in two familial types (also known as early-onset), accounting for less than 5% of all cases, and sporadic (also known as late-onset) with 95% of cases. In the US, approximately 5.5 million people are affected, and the prevalence worldwide is estimated to be as high as 24 million [1]. As life expectancy increases and demographic aging occurs, the global prevalence of Alzheimer’s disease is expected to continue to rise, especially in developing countries [2]. Alzheimer’s disease affects daily life activities and social functioning [3]. The currently available therapeutic approach provides only short-term symptomatic relief but does not slow the progression of the disease. Therefore, the financial costs of Alzheimer’s disease currently exceed USD 185 billion annually in the US, and an additional USD 210 billion worth of unpaid care is provided by friends and family members of patients [3].

The dominant hypothesis that has been held for over 100 years about the etiology of Alzheimer’s disease, and most of the wide-ranging therapeutic struggles in both scientific research and clinical trials, have been built around amyloid and tau protein as the causative agents of the disease [4,5]. However, a causal relationship between amyloid and tau protein and the development of Alzheimer’s disease has not been proven [4,5]. After a comprehensive review of the available data, there is no firm conclusion that amyloid and tau protein play a central or unique role in the etiology of Alzheimer’s disease [4,5]. Regarding two potentially hazardous substances to be blamed for Alzheimer’s disease, recent data show that amyloid and tau protein are triggered by cerebral ischemia, which then interact with each other to produce a synergistic detrimental effect on the neural network that is believed to initiate Alzheimer’s disease [6,7,8,9]. Recent research indicates that Alzheimer’s disease-related proteins and their genes play a key role in the development of post-ischemic brain neurodegeneration with full-blown dementia of Alzheimer’s disease type [6,8,9]. We propose that understanding the etiology of Alzheimer’s disease and implementing a final, causal treatment requires a new approach to this topic, going beyond the current amyloidocentric approach, but without excluding the role of amyloid.

The Janus kinase/signal transducer and activator of transcription (JAK/STAT) signaling pathway are crucial for cell function since several cytokines and growth factors have been identified in the JAK/STAT signaling pathway. It regulates a broad range of cellular processes, including hematopoiesis, immune system functioning, tissue repair, inflammation, apoptosis, and adipogenesis [10]. Therefore, any mutation or loss of JAK/STAT components is related to the pathological condition [11]. JAKs are not covalently associated with cytokine receptors, mediate tyrosine phosphorylation of receptors, and recruit one or more STAT proteins. Tyrosine-phosphorylated STATs dimerize and are then transported into the nucleus through the nuclear membrane to regulate the expression of specific genes [11]. 

Dysregulation of JAK/STAT signaling has been reported following episodes of cerebral ischemia, particularly in experimental studies [12,13,14,15,16]. Yu and Li found that miR-1906 expression was significantly decreased in rats with cerebral ischemia injury [12]. In addition, this overexpression impacted decreased neurological score, infarct volume, brain water content, and the presence of neuronal apoptosis and inflammatory factors (TNF-α, IL-6, and IL-1β) expression. Of note, the phosphorylation of JAK2 and STAT3 was promoted by miR-1906 [12]. Treatment with JAK2/STAT3 pathway inhibitor AG490 inhibited the effects of cerebral ischemic injury by activating the JAK2/STAT3 pathway, which may suggest the association of AG490 and miR-1906 [12,14]. Moreover, studies present the neuroprotective effects of AG490 on cerebral ischemia/reperfusion injury in rats and mice [12,14].

Taking into account the proposed ischemic theory of Alzheimer’s disease, where the possibility of influencing the JAK/STAT signaling pathway has been shown in the brain after experimental ischemia, this target is likely to be used in the prevention or treatment of Alzheimer’s disease [12,13,14,15,16]. In addition, recent clinical data from large real-world datasets together with genome-wide association studies indicate that JAK/STAT signaling is altered in Alzheimer’s disease and is likely to be associated with inflammatory processes in Alzheimer’s disease and has, therefore, been proposed as a target for developing treatments for Alzheimer’s disease [17]. Therefore, JAK/STAT signaling associated with neuroinflammation has been evaluated as a target for drug therapy for Alzheimer’s disease (AD) [17,18]. Still, there is limited data about modulating JAK/STAT signaling pathway in Alzheimer’s disease development; however, the possibility of simultaneously blocking a wide array of pathogenic cytokine production via inhibition of the downstream JAK/STAT pathway is becoming increasingly important. JAK inhibitors are undergoing clinical trials related to autoimmune and inflammatory diseases [19]. The US Food and Drug Administration has already approved five JAK inhibitors (ruxolitinib, tofacitinib, baricitinib, upadacitinib, and fedratinib) to treat some autoimmune/inflammatory and cancer disorders [19]. This increasing number of disorders in which JAK inhibitors are demonstrating efficacy, and the vast pipeline of JAK inhibitors under development make it likely that JAK inhibitors will become crucial in treating autoimmune and inflammatory diseases, including neurological disorders such as Alzheimer’s disease. 

In this review, we set out to highlight the importance of the JAK/STAT pathway during Alzheimer’s disease development and discuss the feasibility of targeting the JAK/STAT pathway as a potential neuroprotective therapy.

## 2. Alzheimer’s Disease

AD is a progressive disease mainly manifesting as slow memory deficits associated with the loss of neuronal cells and synapses. AD is characterized by the abundant presence of amyloid plaques, dystrophic neurites, neurofibrillary tangles (NFTs), and neuropil threads that consist of hyperphosphorylated tau protein [19,20]. Sporadic AD is not considered an inherited disease, but mutations in the genes encoding the amyloid protein precursor (APP), presenilin 1 and 2, and apolipoprotein E ε4 are genetic factors in the development of familial AD [21,22]. 

When it comes to pathological changes in the cerebrovascular system such as ischemic brain infarcts, an increase in amyloid peptide deposition may occur, which can lead to a decline in cognitive function [23]. Decreased blood flow to the brain can result in the deregulation of cyclin-dependent kinase 5 (CDK5) [24], which plays a pivotal role in synaptic plasticity [23,25]. Deregulated CDK5 activity can lead to neuronal cell death in vivo [23,26]. CDK5 is believed to be the primary kinase involved in the hyperphosphorylation of tau protein, leading to neurofibrillary tangles and amyloid plaque development [23,24]. This activity can be found in the post-mortem brains of AD patients [25]. In addition, elevated systemic arterial blood pressure during mid and late-life (40–60 years old) increases the risk of later-life cognitive impairment and AD development [23,27,28].

### 2.1. Modification of Amyloid and Tau Protein in Alzheimer’s Disease

**Figure 1:**
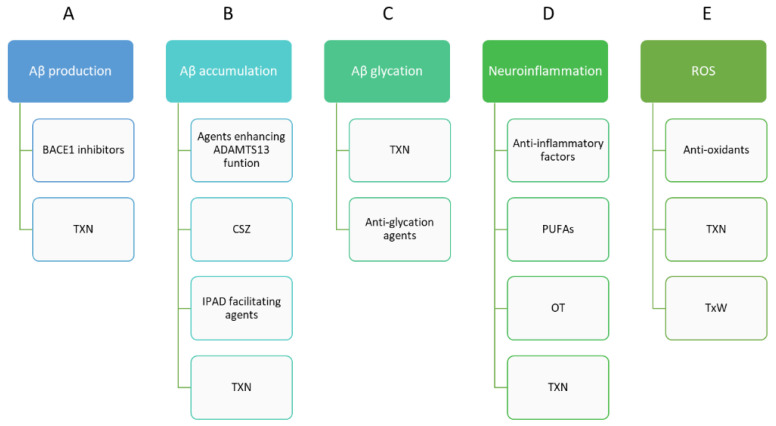
Pathological implications of amyloid-β and its role in potential therapeutic approaches for Alzheimer’s disease. In AD treatment, BACE1 inhibitors inhibit Aβ production (**A**). Another option is taxifolin (TXN) which, with its pleiotropic beneficial effects, suppresses the production and glycation of Aβ; also, it is effective in reducing detrimental Aβ accumulation (**B**). Anti-glycation agents could reduce the accumulation of cytotoxic Aβ oligomers (Aβ glycation) (**C**). Agents that facilitate the formation of mature fibers would also be effective in facilitating the Intramural Peri-Arterial Drainage (IPAD) pathway or enhancing ADAMTS13 function. Anti-inflammatory mediators (**D**) and antioxidants (**E**) and may also exhibit protective effects against AD. Aβ, amyloid-β; AD, Alzheimer’s disease; ADAMTS13, a disintegrin and metalloprotease with thrombospondin type I motif, member 13; BACE1, β-site amyloid precursor protein cleaving enzyme-1; CSZ, cilostazol; OT, oxytocin; PUFAs, polyunsaturated fatty acids; ROS, reactive oxygen species; TwX, Twendee X; TXN, taxifolin.

The hallmark depiction of AD is the abundant presence of amyloid plaques and neurofibrillary tangles consisting of hyperphosphorylated tau protein [19,20]. Amyloid plaques are extracellular deposits of Aβ; they are 40 or 42 amino acids long and derived from the APP [29]. These plaques can be found localized to the layers of the neocortex [20]. Their origin is unknown but has been hypothesized to form from glial amyloid secretion, soma and synaptic Aβ peptide secretion from neurons, or dystrophic neurites [19,21]. As the disease progresses, amyloid plaques develop from a diffuse form to a dense/senile form [23]. They consist of a central core comprised of a 4-kD peptide with a βA4 beta-pleated sheet configuration [23]. The Aβ peptide is derived from a highly conserved transmembrane glycoprotein, an amyloid protein precursor [30,31,32]. Their central core is encircled by changed neurites that are comprised of groups of paired helical filaments, dense bodies, and membranous profiles [23]. It is not completely understood why some amyloid peptides can remain in a diffused state while others aggregate into a highly dense form [19]. Studies of post-mortem transgenic mice with Alzheimer’s disease revealed that plaques are initially small and then become more numerous and increase in size [19,22]. Initial plaque formation is very fast, but as the disease progresses, growth becomes more gradual, and the plaques then cluster together to form larger plaques [19,22]. Furthermore, large plaques can also contain multiple dense cores that have fused over time [22]. So-called “flower plaques” morphology can appear from new plaques that form over time in close vicinity of previous plaques and cluster together [22,33]. The potential therapeutic approach for AD is presented in Figure 1.

A definitive diagnosis of Alzheimer’s disease is made after a neuropathological examination of the brain post-mortem [23]. This is accomplished by identifying characteristic neuropathological changes in brain tissue [23]. NFTs are abnormal fibrous inclusions found in the cytoplasm of pyramidal neurons, most commonly in the hippocampus, entorhinal cortex, and isocortex [31,34]. These abnormal fibrils are at first presented in pairs and are helically wound [31,35]. The abnormally phosphorylated microtubule-associated protein tau is the main component of NFTs [23,26]. Putative AD-specific proteins (A68) were shown to be the main subunit of paired helical filaments [36]. The core of A68 was found to be indistinguishable from the tau protein through amino acid sequencing and immunological data, where antibodies were directed against the abnormally phosphorylated tau protein [31,36]. Other proteins, such as ubiquitin, cholinesterase, and amyloid protein precursor, were also found to be associated with NFTs [31]. The distribution and extent of NFTs have a direct correlation to dementia, along with the duration and impact of the disease [31,37]. This means that NFTs are directly involved in the health and functionality of the brain [23].

Other commonly found pathological lesions are vascular amyloid deposits found in the cerebral cortical vessels, called cerebral amyloid angiopathy [23]. Peptide accumulation can be found in the walls of small arteries and leptomeninges arterioles [23]. The peptide accumulation does not clog the vessel, nor does it interfere with vesical function [23]. However, spontaneous vascular rupture is common, leading to the pooling of blood in the brain tissue [23]. Also known as a lobar hemorrhage or micro bleeding, these events are rare in AD and only account for a scarce number of fatal intracerebral complications [23].

Granulovacuolar degeneration bodies, a poorly understood type of lesion, consist of intraneuronal clusters of small vacuoles that contain a dense basophilic granule [31]. These small vacuoles measure 2 to 4 μm in diameter [23]. They are almost exclusively found in pyramidal neurons of the hippocampus [23]. These double membrane-bound structures contain an electron-dense central granule along with a clear vacuolar matrix [31,38]. This signature morphology bears a similarity to autophagy organelles [39]. Autophagy marker proteins were identified as correlated to late-stage organelle maturation, more precisely after autophagosome formation but before autolysosome maturation was complete [38]. An accumulation of those bodies occurs at the nexus between endocytosis and autophagy, the two major pathways in neurons to the lysosome [38]. Furthermore, granulovacuolar degeneration bodies accumulation correlated directly to the failure to complete autolysosomal formation [38]. Granulovacuolar degeneration is not disease-specific and can be found in other neurodegenerative disorders, as well as in elderly people with no neurological symptoms [38]. Although not well understood, granulovacuolar degeneration was commonly found in between the CA1 and CA2 regions of the hippocampus, assisting in diagnosing AD [31,40].

Additionally, Hirano bodies, which are eosinophilic rod-like perineuronal lesions, were found in the CA1 section of the hippocampus [23]. They have a tweed fabric appearance, consisting of parallel fibers that interweave in a crossing pattern [31,41]. These parallel fiber bodies consist of actin, tropomyosin, and vinculin [31,41]. The presence of these fiber bodies indicates that Hirano bodies are derived from the atypical organization of the neuronal cytoskeleton [41]. Tau protein is known to have an affinity to F-actin in the neuronal cytoskeleton [41]. This alteration leads to the formation of Hirano bodies and plays an important role in the progression of the disease. An interaction is observed between the tau protein and amyloid protein precursor intracellular domain that leads to the activation of kinase GSK3β, causing the promotion of cellular death [42]. These lesions can be found in AD patients, but they also may be encountered in the brains of healthy subjects with normal cognition [23].

Though not one of the above lesions, a significant amount of synaptic loss has been observed in patients with AD [31,43]. Studies showed that patients with AD had a 45% loss of presynaptic proteins [23]. Such a significant loss of neuronal communication leads to neuropathological changes and loss of cognitive function with the final development of full-blown dementia [31,44].

### 2.2. The Role of Innate Immunity in Alzheimer’s Disease 

The main immune cells of the innate immune system of the brain are microglial cells [45,46]. A high concentration of microglial cells can be found in the basal ganglia and hippocampus [45,46]. These macrophages of the brain are continuously working, always looking for loose cellular debris or pathogens to eliminate [45,47]. On the surface of the microglial membrane, there are many receptors that mediate signaling, such as cytokine and chemokine receptors, pattern-recognition receptors, and toll-like receptors (TLRs) [45,47], which maintain cerebral homeostasis, neuroprotection, synaptic protection, plasticity, and neurogenesis [45,46]. Microglia secrete neurotrophin 3 and 4, brain-derived neurotrophic factor (BDNF), and nerve growth factor, which promotes neural plasticity and protection [45,47]. The release of BDNF increases neuronal tropomyosin receptor kinase B phosphorylation, a key mediator of neuronal survival, differentiation, and plasticity [48,49]. BDNF was detected not only in microglia but also in oligodendrocytes and astrocytes [48,50]. Microglial BDNF has been shown to play an important role in the brain of healthy individuals, creating learning-induced synapses [48,51].

Activation of the innate immune system is associated with triggering receptors expressed on myeloid cells 2 (TREM2) found in microglial cells, osteoclasts, macrophages, and dendritic cells [45,52]. The TREM2 protein inhibits the production and secretion of inflammatory cytokines released from microglia and, as a result, suppresses the inflammatory response [45]. Moreover, TREM2 regulates the phagocytic process and the removal of neuronal debris and amyloid deposits [45,53].

R47H, a functional variant in the TREM2 gene, leads to reduced function and an increase in AD susceptibility [45]. Loss of function of TREM2 leads to a reduced phagocytic ability to remove amyloid plaques, consequently leading to AD pathology [45,52]. Inversely, the overexpression of TREM2 leads to enhanced phagocytic capability [45,54]. 

Another regulator of cytokine secretion and endocytosis of amyloid deposits is the transmembrane glycoprotein cluster of differentiation 33 (CD33) [45,55]. AD patients show an increased level of CD33 proteins in microglial cells [45,56]. On the other hand, rs3865444, a CD33 single-nucleotide polymorphism, was suggested to be the reason for late-onset Alzheimer’s disease [45,57]. Moreover, the rs3865444C allele proved to lead to an abundance of amyloid plaques and an increase in AD [45,58]. Conversely, the amyloid plaques were significantly reduced for the rs3865444A allele, reducing the risk for AD [45,58]. 

Toll-like receptors, which can be found on the microglial membrane surface, recognize invading pathogens and damaged cells and activate signaling molecules that lead to cytokine release. TRLs play a major role in maintaining CNS homeostasis [45,59]. TLR2, TLR4, and TLR9 all had implications for the pathogenesis of AD [51]. TLR2 had both a positive and negative effect, promoting amyloid deposit uptake by the microglia, as well as releasing pro-inflammatory cytokines leading to neuronal damage [45,60]. But for TLR9, only a beneficial role of amyloid plaque removal is observed during the progression of AD [45]. TLR9 activation led to increased microglial phagocytosis, reducing amyloid plaque accumulation [45,61]. Additionally, a reduction in NFTs was also observed from TLR9 activation [45,62].

### 2.3. Neuroinflammation

Studies have shown that neuroinflammation plays a significant role in the pathogenesis of AD [46]. Several cells of the immune system have been implicated in the pathogenesis of AD, including microglial cells, astrocytes, and some peripheral inflammatory cells such as macrophages [63,64,65,66,67].

The microglia play a pivotal role as macrophages of the central nervous system, in providing protection to neurons and in synapse remodeling. They provide protection to neurons and function in the remodeling of synapses. Microglia are beneficial and detrimental in AD [48]. They are responsible for clearing the CNS of pathogenic activity and restoring homeostasis to the CNS [49]. There are two major responses of microglia to tissue damage, M1, which is pro-inflammatory, leading to the activation of interleukin-1 (IL-1), IL-6, and tumor necrosis factor-alpha (TNF-α). M2 is anti-inflammatory and leads to the activation of IL-4, IL-10, and IL-13 [47]. The restorative activity of microglia is also believed to play a part in neurodegeneration in AD [50]. The proposed auto-toxic loop hypothesized that the activation of microglia in response to tissue damage or amyloid deposition leads to further tissue damage, which in response also causes the activation of more microglia resulting in a cycle of tissue damage and microglia activation [47]. In AD, microglia binds to soluble APP via receptor Class A scavenger A1/toll-like receptor, which can stimulate the activation of the inflammatory process leading to further tissue damage and APP processing [47,48]. Moreover, increased cytokine levels are responsible for insufficient phagocytic activity on injured neurons and oligodendrocytes [47]. On the other hand, in aging CNS, microglia have enhanced sensitivity to inflammatory stimuli leading to a more pronounced neuroinflammatory response and tissue damage [47]. The positive feedback between APP formation and inflammatory response leads to the production of reactive oxygen species, causing further tissue damage [48].

Astrocytes play several roles in the CNS, including regulation of the transmission of electrical impulses and maintenance of the blood–brain barrier [49]. They are also known to play a role in the development of AD by promoting the inflammatory response through the release of interleukins, nitric oxide, and other cytotoxic molecules when exposed to APP [63,64,67].

### 2.4. The Canonical JAK/STAT Signaling Pathway

The Janus kinase/signal transducer and activator of the transcription signaling pathway were first discovered in 1990 when studying how interferon (IFN) leads to the activation of a transcription factor [68]. The JAK/STAT signaling cascade consists of three main components: a cell surface receptor, a Janus kinase (JAK), and two signal transducers and activator of transcription (STAT) proteins. There are four members of the JAK family: JAK1, JAK2, JAK3, and TYK2, which consists of non-receptor tyrosine protein kinases. When cytokines bind to their receptors, JAK tyrosine kinases are activated and transmit regulatory signals [11]. Activated JAKs then phosphorylate tyrosine residues on the receptor, creating binding sites for proteins with SH2 domains. SH2 domain-containing STATs are recruited to the receptor, where they are also tyrosine-phosphorylated by JAKs. JAK family members are expressed in almost all tissues [69], except JAK3, which is expressed in the bone marrow, lymphatic system, and endothelial and vascular smooth muscle cells [70]. The STAT family comprises seven members: STAT1, STAT2, STAT3, STAT4, STAT5a, STAT5b, and STAT6 [11], which consist of 750–900 amino acids. From the N-terminus to the C-terminus, there are the N-terminal domain and coil, helix domain, DNA-binding domain, connection domain, SH2 domain, and transcription-activation domain. Six domains regulate different functions of STAT. STAT4, STAT5, and STAT6 can be used as targets for ubiquitin-dependent destruction, while STAT1, STAT2, and STAT3 are more stable, indicating that the transcriptional active region also regulates protein stability [11]. STATs are latent transcription factors that reside in the cytoplasm until activated. These activated STATs form hetero- or homodimers and translocate to the cell nucleus, where they induce transcription of target genes. The phosphorylated sites on the receptor and JAKs serve as docking sites for the SH2-containing STATs, such as STAT3, and for SH2-containing proteins and adaptors that link the receptor to MAP kinase, PI3K/Akt and other cellular pathways.

**Figure 2:**
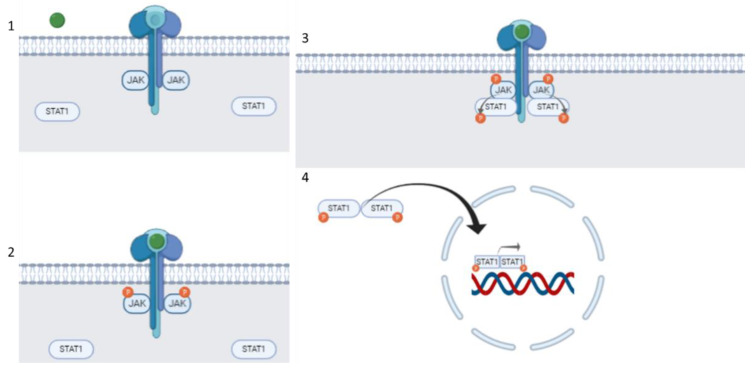
JAK/STAT signaling pathway. The cell ligand interacts with its receptor to cause receptor dimerization. The connection between the ligand and the receptor induces transphosphorylation of JAK. Activated JAK causes tyrosine phosphorylation of the bound receptor, forming a docking site for STATs. In the next step, JAK phosphorylates STAT, and then STAT dissociates from the receptor and forms homodimers or heterodimers through SH2-domain–phosphotyrosine interactions. These dimers translocate to target gene promoters, regulating the transcription of the target genes [11,72]. Steps are as follows: (1) Ligand binds to the receptor, (2) JAK auto-phosphorylates, (3) STAT binds to JAK and JAK phosphorylates STAT, (4) STAT forms homo-dimer; therefore, the homo-dimer enters the nucleus, binding to DNA, targeting gene transcription.

The JAK/STAT signaling pathway transmits information from extracellular chemical signals regulating growth, survival, differentiation, and pathogen resistance. Notably, the normal function of this pathway is essential for neural stem cell maintenance [71]. The mechanism of action of JAK/STAT signaling pathway is shown in Figure 2.

Disrupted or dysregulated JAK/STAT functionality can result in inflammation, immune deficiency syndromes, cancers, and neurodegenerative diseases [71]. The cell surface receptors and cytokines, such as interferon, interleukin, and growth factors, activate associated JAKs, increasing their kinase activity. 

### 2.5. Regulation of JAK/STAT Signaling Pathway

JAKs are most commonly regulated via various post-translational mechanisms [73]. One such example is the suppressor of cytokine signaling (SOCS) proteins which are regulators of JAK/STAT signaling [73]. The SOCS protein family contains eight members: SOCS1–SOCS7 and CIS (cytokine-inducible SH2 domain protein) [73,74]. There is a conserved SH2 domain found throughout the SOCS family [73]. SOCS proteins are rapidly induced by cytokines, leading to the inhibition of the JAK/STAT signaling pathway, forming a negative feedback loop [73,75]. Through this direct mechanism, SOCS proteins inhibit cytokine signaling [73]. SOCS1 binds to tyrosine-phosphorylated JAKs at the SH2 domain, leading to direct inhibition of JAK activity [73,76]. SOCS3 is required to bind to the activated JAK receptor in order to inhibit JAK [73,77]. SOCS1 and SOCS3 were considered essential in the regulation of immune function [73]. Both are induced by interferon-γ and IL-6, and both can be inhibited by interferon-γ and IL-6 [69]. SOCS proteins have a specific effect on the kinetics of the JAK/STAT complex [73]. In the absence of SOCS, cytokines may induce a prolonged activation of STAT, leading to the possibility of an altered cytokine response [69]. As for CIS, these proteins do not directly act on JAK but act slightly differently, outcompeting STAT to bind to its own receptor, inhibiting STAT activation [73,78].

Another example of regulators of JAKs is protein tyrosine phosphatases (PTPs) [73]. There are several PTPs involved, including SHP1, SHP2, CD45, PTP1B, and T cell PTP [73]. Similar to SOCS proteins, SHP1 and SHP2 contain a conserved SH-domain [73]. SHP1, expressed by hematopoietic cells, is associated with the β-chain of the IL-3 receptor and erythropoietin receptor, as well as the dephosphorylation of JAK1 [73], whereas SHP2 participates in the negative regulation of JAK1 [70]. CD45 is a PTP receptor that is also expressed in hematopoietic cells and plays a crucial role in T cell and B cell antigen-receptor signaling [73,79]. CD45 functions by directly binding and dephosphorylating all JAKs [73,80]. Deletion of CD45 leads to increased formation of erythroid colonies, which is consistent with its role in erythropoietin and IFN regulation [69]. Lastly, PTP1B and TCPTP are two related PTPs whose mechanism is to dephosphorylate JAKs [73]. PTP1B specifically targets JAK2 and TYK2, but not JAK1 [73]. PTP1B is shown to negatively regulate leptin signaling via targeting JAK2 [73,81]. TCPTP, like many PTPs, can dephosphorylate JAK1 and JAK3 proteins, reminding us of their importance in the regulation of cytokine signaling.

Another pathway that has been implicated in the regulation of JAK/STAT signaling is ubiquitylation [73]. Efficient ubiquitylation is achieved once JAK2 has been phosphorylated with tyrosine [73]. Polyubiquitylated JAK2 can then be degraded. Co-expression with SOCS1 further promotes the degradation of JAK2 [73]. Meaning JAK2’s stability is regulated by the SOCS1-mediated ubiquitin–proteasome pathway [69].

When it comes to STATs, they can be regulated via post-translational modification by phosphorylation, acetylation, methylation, ubiquitylation, and tyrosine phosphorylation [73]. Cytokine receptor stimulation leads to the phosphorylation of STATS at a conserved tyrosine residue. This leads to STATs’ dimerization, entrance into the nucleus, and DNA binding [69,81]. As a result, tyrosine phosphorylation is viewed as an activating switch for STAT [73].

STAT1 can be methylated by the protein arginine methyltransferase 1 at Arg31 and has been found to be associated with IFN-α/β receptors [73,82]. STAT1 methylation leads to an increase in DNA-binding activity [73,82]. Phosphorylation of STAT1 occurs at Ser727, a mitogen-activated protein kinase (MAPK) site, which suggests the need for induction of IFN genes [73]. Several kinases are linked to STAT phosphorylation, including p38, extracellular signal-regulated protein kinase, JUN N-terminal kinase, and protein kinase Cδ [73,82]. STAT6 has been known to be involved in acetylation through histone acetyltransferase cAMP response element-binding protein (CREB)-binding protein (CBP)/p300, inducing transcriptional activation of the 15-lipoxygenase-1 gene activation [70,83].

STATs can also be post-translationally modified via ubiquitylation [73]. Certain members of the Paramyxovirus family of RNA viruses encode a “protein V”, which stimulates polyubiquitylation and leads to proteasomal degradation of STATs. For example, the Type II Human Parainfluenza virus directly targets STAT2-inducing degradation. The mumps virus also induces the degradation of STAT1 and STAT3 [69,84,85,86,87].

Another method of STATs regulation is by the PIAS family, also known as Gu-binding proteins [73,84]. The mammalian PIAS family contains four members: PIAS1, PIAS3, PIASX, and PIASY [73,83]. In the central region of the PIAS protein, there is a highly conserved region called a ring-finger-like zinc-binding domain [73]. In vivo study of cultured mammalian cells showed a PIAS-STAT interaction after cytokine stimulation [73]. PIAS1, PIAS3, and PIASX showed specific interaction with STAT1, STAT3, and STAT4 [73,84]. PIAS and STAT interaction is cytokine-dependent and does not interact otherwise [73]. If overexpressed, PIAS3 interacts with STAT5 regulating prolactin-mediated gene expression [73,85]. Each member of PIAS has been shown to inhibit STAT, regulating gene expression [73]. PIAS1 and PIAS3 are both able to inhibit DNA binding of STAT1 and STAT3 [73,84], whereas PIASX and PIASY can inhibit STAT4 and STAT1-dependent transcription without affecting their actual binding activity to the DNA [73]. Furthermore, members of the PIAS family have also been shown to enhance transcriptional factors, such as androgen receptors, but how this mechanism works is yet to be understood [73]. 

Protein tyrosine phosphatases are also involved in the regulation of STATs in both the nucleus and cytoplasm [73]. SHP2, located in the cytoplasm, is responsible for the dephosphorylation of STAT5 [73,86]. SHP2 is also responsible for the dephosphorylation of STAT1 at tyrosine and serine markers [69]. PTP1B is known to dephosphorylate STAT5 but only when overexpressed [73,87]. However, establishing whether STAT5 is a physiological substrate of PBP1B has yet to be achieved [73]. When it comes to regulatory activity within the nucleus, PTP was shown to inactivate STAT1 [73]. Biochemical purification methods were used to identify TC45, which can directly dephosphorylate STAT1, rendering it inactive [73,88].

Cross-regulation among the STAT family by other STAT proteins was identified to play a key role in cytokine-signaling specificity [73]. STATs form homo- or hetero-dimers, producing three possibilities of DNA-binding activity, STAT1-STAT1, STAT3-STAT3, and STAT1-STAT3 [73]. IL-6 activates both STAT1 and STAT3, and the removal of STAT3 in mice resulted in prolonged STAT1 activity, initiating an IFN-γ-like response [73,89]. Therefore, the IL-6 signaling specificity of STAT3 shows communication between STAT1 and STAT3.

### 2.6. The JAK/STAT Signaling Pathway during Alzheimer’s Disease Progression

Microglia-mediated neuroinflammation is one of the most remarkable hallmarks of neurodegenerative diseases. Microglia-induced neuroinflammation contributes to the pathogenesis of AD through direct damage to the neuron, concurrently promoting protein aggregations, suggesting that it should be a new target for AD treatment [90]. 

Chiba et al. pointed out that STAT3 inactivation is involved in the pathogenesis of AD [91]. They found that the phosphorylated (p-) or activated form of STAT3 (p-STAT3) was significantly reduced in hippocampal neurons of clinically and post-mortem diagnosed AD patients compared to the control group [91]. Since amyloid species presence is a characteristic feature of AD, amyloid may be a crucial cause of STAT3 inactivation [91]. Another correlation is observed between p-STAT3 immunoreactivity in hippocampal neurons of young subjects compared to older normal subjects in both humans and rodents and endogenous levels of insulin growth factor-1 activating STAT3 [92]. Levels of both are decreased with aging, suggesting that this decrease may be linked to the pathogenesis of AD [93]. Given that disturbed STAT3 activity by aging and the neurotoxic amyloid causes memory impairment related to AD, STAT3 activation will provide a novel therapeutic strategy for AD. Therapeutic mechanisms of STAT3 activation seem to involve the enhancement of cholinergic neurotransmission [91].

Several cellular systems are involved in the prevention of uncontrolled neuroinflammation. The SOCS proteins are one such system responsible for enhancing the degradation of activated receptors and removing the stimuli responsible for receptor activation [94]. When it comes to AD, chronic neuroinflammation is a fundamental pathological feature that occurs in response to amyloid and tau protein pathology [94]. SOCS1 and SOCS3 are two of the most studied regulatory proteins, as they are known to modulate inflammatory signaling in monocytes and astrocytes [94]. When expressed in human neurons, SOCS1 inhibited IFN-γ induction of the MHC-11 protein. SOCS3, on the other hand, inhibited insulin growth factor-1 generated neurite growth [94,95]. 

### 2.7. JAK/STAT as a Potential Therapeutic Target in Alzheimer’s Disease

Since the JAK/STAT signaling pathway is involved in extracellular cytokine-activated receptor-mediated signal transduction, associated with cellular proliferation and differentiation, organ development, and immune homeostasis, it may be a potential target treatment in various neurodegenerative diseases, including Alzheimer’s disease.

Drug therapy that targets the JAK/STAT signaling pathway can be classified into three types: (i) cytokine or receptor antibodies, (ii) JAK inhibitors, and (iii) STAT inhibitors. 

Since Alzheimer’s disease is characterized by neuronal loss mainly in the hippocampus with no known etiology, and cerebral ischemia is characterized by the same pathological changes as AD, ischemia has now been proposed as an etiological factor in AD [8,96]. Given the importance of cytokines in regulating all phases of the immune response, the successful generation of a selective JAK inhibitor demonstrates the feasibility of targeting JAKs and raises the question of whether targeting other JAKs might also be useful. Treatment with JAK2/STAT3 inhibitor AG490 resulted in the phosphorylation of JAK2, and STAT3 was inhibited, and the neurological score, brain water content, neuronal apoptosis, and inflammatory factors were reversed, suggesting a protective response to cerebral ischemic injury through activating the JAK2/STAT3 pathway [12,14]. Another protective effect against cerebral ischemia injury is Shaoyao-Gancao (SGD) [16,97]. The SGD and its active components, including paeoniflorin, glycyrrhizin, and liquiritin, play neuroprotective roles by inhibiting inflammatory responses in neurological disorders. Additionally, it has been shown to be effective in learning and cognitive improvements by decreasing neuroinflammation in ischemic brain injury and Alzheimer’s disease [16,97].

#### 2.7.1. Janus Kinase Inhibitors (Jakinibs)

Numerous cytokines underlie the pathogenesis of AD; thus, they are potential targets of therapy. This has led to the development of monoclonal antibodies and recombinant proteins that target these cytokines and their receptors. One such class of drugs that were developed for the inhibition of cytokine signaling is called Janus kinase inhibitors (Jakinibs). These inhibitors are essential signaling mediators downstream of various known pro-inflammatory cytokines and, thus, are being used as an effective method to treat pathologies due to inflammatory disorders [98]. For example, IL-6 is a pro-inflammatory cytokine and is commonly overexpressed in many autoimmune diseases, as well as being a driver of acute phase response induction of serum amyloid A [98].

The pathological basis of AD has been identified as neuronal apoptosis and synaptic dysfunction or loss. These damages can accumulate from an increase in amyloid generation, an increase in iron deposits, and oxidative stress [99]. It has been found that Kruppel-like factor 4 (KLF_4_) plays a crucial role in CNS regulation. KLF_4_ is a zinc finger-containing nuclear protein that regulates gene transcription [99]. KLF_4_ is known to regulate neuronal apoptosis, synaptic regeneration, and neuroinflammation [99]. Early loss of axons is a common feature in neurodegenerative diseases [99]. Studies have confirmed that chronic oxidative stress can disrupt the balance of amyloid peptides by enhancing the expression of phospholipase A2 group 3 (Plas2g3) in astrocytes, subsequently leading to the development of AD [99,100]. KLF_4_ plays an important role in inhibiting the onset of oxidative stress-induced apoptosis [99,101]. 

KLF_4_ can affect axon regeneration through JAK/STAT3 pathway [99]. A decrease in KLF_4_ expression led to an increase in STAT3 phosphorylation, regulating axonal growth [99,102]. However, an increase in KLF_4_ would lead to no STAT3 binding to DNA, which improves axonal regeneration [99,103]. KLF_4_ may be targeted as a therapeutic agent against oxidative stress [99]. Though the mechanism remains unclear, drugs such as statins can activate a mitogen-activated protein kinase 5, leading to the expression of KLF_4_ [99,104]. Thus, it can be assumed that KLF_4_ positively regulates neuronal apoptosis and negatively affects axonal repair [99].

KLF_4_ is a known regulator of neuroinflammation via the mediation of macrophage release. Chronic inflammatory reactions in the CNS are associated with an increase in KLF_4_ expression, inducing neuroinflammation [99,105]. Silencing KLF_4_ restricts neuroinflammation. Under neuroinflammatory conditions, amyloid plaques accumulate, leading to the activation of astrocytes and microglia [99,105]. Astrocytes, in turn, further enhance neuroinflammation with the release of pro-inflammatory factors IL-1, IL-6, and TNF-α [99,106]. This continuous inflammation eventually results in dysfunction and neuronal apoptosis, the onset of AD [99].

As regards iron beyond the normal physiological range, this can lead to an accelerated formation of amyloid plaques and hyperphosphorylated tau protein, which increases oxidative stress and leads to AD [99,107]. It has been shown that stress induces glucocorticoid release, which in turn activates the KLF_4_- Heme Carrier Protein 1 pathway, which increases the uptake of heme [99,108]. This, in turn, leads to the accumulation of iron in the brain, worsening the brain damage [99,108]. 

To conclude, KLF_4_ is responsible for anti-apoptosis, anti-inflammation, axon regeneration, and iron accumulation in the CNS, which all play a role in the development of AD [109]. This suggests exploring KLF_4_ as a potential therapeutic target for AD [109]. Therapeutic modulators of STAT are another potential method for targeting AD [109]. Negative regulators such as suppressors of cytokine signaling and protein tyrosine phosphatases down-regulate STAT signaling [109].

#### 2.7.2. JAK Inhibitors

JAK inhibitors are a group of small-molecule inhibitors with different chemical structures [11]. Their therapeutic effect is due to immunosuppression and reduction of the abnormally elevated serum pro-inflammatory cytokines mediated by the JAK/STAT signaling pathway. Tofacitinib and baricitinib are the first orally available JAK inhibitors to be approved for treating autoimmune diseases [110].

#### 2.7.3. STAT Inhibitors

These methods include STAT dimerization inhibition using peptidomimetics or peptides, small molecules, natural products, virtual or library screening, and inhibition of tyrosine kinase [111]. Most research has focused on the pTyr-SH2 domain interaction, which is key to promoting STAT3 dimerization [111]. Peptide inhibitors that target the pTyr-SH2 domain interaction were the first STAT3 inhibitors [111,112]. Peptides and peptidomimetics disrupt the STAT3 pTyr-SH2 domain interaction and STAT3 dimerization [111,112]. Analogs such as ISS-610 and S3I-M2001 showed effectiveness against STAT3 activity in vitro [111,113]. Cell growth inhibition and induced apoptosis were both seen in vitro with ISS-610 and S31-M2001 [111,113].

Computational modeling, docking studies, and virtual screening of chemical libraries were used to identify various small-molecule inhibitors of STAT3 activity. STA-21, a known small-molecule inhibitor, disrupts STAT3-STAT3 dimerization [111]. Another non-peptide small molecule that targets the STAT3 SH2 domain is stattic [111,114]. Stattic inhibits STAT3 signaling, inducing apoptosis [114]. The next method of STAT inhibition is through oligonucleotide (ODN) decoys which compete with the promoter to bind to the transcription factor, preventing gene expression [111]. A study by Hückel et al. [115] involving a cardiac allograft model of atherosclerosis in a rat used a 25-bp STAT1 ODN specifically down-regulated CD40 levels, which is a STAT1 target gene. This led to T cell activation and CD40 suppression by STAT1 ODN, presenting a protective effect against atherosclerosis advancement. In a study by Shen et al., a 15-bp STAT3 ODN decoy (5′-CATTTCCCGTAAATC-3′) was used to evaluate glioblastoma xenografts in mice [116]. This led to a down-regulation of STAT3-associated genes and decreased tumor growth. The same ODN decoy (5′-CATTTCCCGTAAATC-3′) was used in a study by Sen et al. to inhibit STAT3 function against resistant head and neck squamous cell carcinoma and bladder cancer [117].

Another approach used to attempt to modulate STAT3 is the use of antisense oligonucleotides (ASOs). In vitro melanoma and mammary carcinoma models were used and showed a down-regulation of STAT3 by specific ASOs, which inhibited gene expression and down-regulated the STAT3 target gene vascular endothelial growth factor [118].

A therapeutic study was then attempted using ASOs to inhibit STAT function. ISIS Pharmaceuticals (ISIS 481464) designed a phosphorothioate-modified chimeric ASO sequence that would target STAT3 mRNA. Cynomolgus monkeys were used as the model and were given a 10 mg/kg per week dose for 6 weeks. This resulted in STAT3 levels decreasing by up to 90%. When given a 30 mg/kg per week dose for 6 weeks, no treatment-related changes or deaths were seen, and the larger dose was well tolerated [109].

Currently, cholinesterase inhibitors (ChEIs) such as donepezil, rivastigmine, and galantamine are clinically used as first-line drugs for AD, although the clinical effectiveness of these drugs is still controversial [119]. ChEIs inhibit ChE and increase ACh concentrations in the synaptic clefts to potentiate cholinergic neurotransmission. The STAT3-activation therapy also supports cholinergic neurotransmission through distinct mechanisms: upregulation of ChAT and M1 mAChR sensitization, combined with the fact that STAT3 activation contributes to neuronal survival [91]. In addition, memantine, an inhibitor of the NMDA receptor, is also approved for AD treatment [120]. Memantine is used to alleviate Glu-induced excitotoxicity via the NMDA receptor. 

#### 2.7.4. Natural Products and Derivatives

Curcumin is a naturally occurring nutraceutical compound found in the rhizome plant Curcuma longa. Curcumin inhibits STAT3 and diminishes the expression of STAT3 and STAT6 by up-regulating SOCS1, SOCS3, and PIAS3 [121]. Clinical data suggest that dietary intake of curcumin enhances neurogenesis and offers neuroprotection against APP and tau protein pathology [122]. 

Another natural component is resveratrol, a polyphenolic stilbenoid found in grapes, mulberries, peanuts, rhubarb, etc. This substance targets inflammatory cytokines, nuclear factor-κB, sirtuin, adenosine monophosphate kinase, and antioxidant enzymes at the molecular level. In addition, resveratrol regulates immune responses by suppressing the phosphorylation of STAT1, STAT3, and NF-κB signaling pathways [123].

Myricetin is a flavonoid present in vegetables, fruits, nuts, berries, herbs, and plants together with beverages (tea, wine) that displays antimicrobial, antioxidant, antidiabetic, anticancer, immunomodulatory, and neuroprotective activities [124]. It has a positive impact on the body by affecting multiple cell systems and modulating the activity of various pathways to reduce cognitive decline and neuronal dysfunction [125]. The neuroprotective effects of this compound in Alzheimer’s disease reduce the production of Aβ due to the inhibition of BACE-1 activity to digest APP. In addition, the MYR reduces the production of Aβ by increasing the level of α-secretase and competitively decomposing APP and inhibiting the β-sheet by binding to Aβ, and preventing the conversion of Aβ monomers into oligomers and fibrils [126]. In addition, myricetin protects against the same neuropathological changes in the ischemic brain as in AD [126].

## 3. Conclusions

The role of neuroinflammation in AD is apparent as a key factor. Clinical studies show that characteristic inflammatory proteins occur in blood patients with AD [127,128]. Moreover, post-mortem studies present that inflammatory response is associated with AD neuropathology [129,130]. In addition, there is increasing evidence from genome-wide association studies and rare mutations that genes encoding proteins involved in immunity are amongst the most consistently associated with AD [131]. Treatment with non-steroidal anti-inflammatory drugs can decrease the risk of AD development [132]. 

The Janus kinase/signal transduction and activator of the transcription pathway transduce downstream of multiple cytokines, chemokines, and microglia activation [133,134], critical to the pathogenesis of immune-mediated disease and neurodegenerative disorders. Dysregulation of the JAK/STAT signaling pathway is recognized as a major contributor to various diseases, particularly Alzheimer’s disease. Experimental, clinical, and genomics data indicate that the JAK/STAT signaling pathway can be a potential target for therapy. In conclusion, this is a novel pathway accounting for pathological dysfunction in AD, such as amyloid- and age-dependent inactivation of the JAK2/STAT3 axis in the hippocampal neurons causing memory impairment related to AD by disturbing cholinergic neurotransmission. In addition, STAT3 inactivation causes cholinergic dysfunction via dual mechanisms, presynaptic downregulation of cholinergic genes such as ChAT and postsynaptic desensitization of M1 mAChR. Notably, the functional deterioration in the JAK2/STAT3 axis AD models can be reversed, concomitantly with memory improvement through activation of the JAK2/STAT3 axis without affecting amyloid levels. Future studies should focus on extensive insight into the underlying mechanisms of the JAK/STAT pathway effects and development of Alzheimer’s disease. This approach would be helpful in exploring and finding potential biomarkers of Alzheimer’s disease and/or efficient therapeutic targets with fewer side effects.

## Data Availability

Not applicable.

## Abbreviations

AD	Alzheimer’s disease
ADAMTS13	Disintegrin and metalloprotease with thrombospondin type I motif, member 13
APP	Amyloid protein precursor
ASOs	Antisense oligonucleotides
Aβ	Amyloid-β
BACE1	β-site amyloid precursor protein cleaving enzyme-1
BDNF	Brain-derived neurotrophic factor
CDK5	Cyclin-dependent kinase 5
CHEIs	Cholinesterase inhibitors
CSZ	Cilostazol
IFN	Interferon
IL-6	Inteleukin-6
IPAD	Intramural Peri-Arterial Drainage
JAK	Janus kinase
JAK/STAT	Janus kinase/signal transducer and activator of transcription signaling pathway
KLF_4_	Kruppel-like factor 4
MAPK	Mitogen-activated protein kinase
NFTs	Neurofibrillary tangles
ODN	Oligonucleotide decoys
OT	Oxytocin
PTPs	Protein tyrosine phosphatases
PUFAs	Polyunsaturated fatty acids
ROS	Reactive oxygen species
SOCS	Suppressor of cytokine signaling
STAT	Signal transducers and activator of transcription
TLRs	Toll-like receptors
TNF-α	Tumor necrosis factor-alfa
TREM2	Triggering receptors expressed on myeloid cells 2
TwX	Twendee X
TXN	Taxifolin
